# Exploration of the Structure and Recognition of a G-quadruplex in the her2 Proto-oncogene Promoter and Its Transcriptional Regulation

**DOI:** 10.1038/s41598-019-39941-5

**Published:** 2019-03-08

**Authors:** Xiaojie Cui, Han Chen, Qiang Zhang, Ming Xu, Gu Yuan, Jiang Zhou

**Affiliations:** 1 Beijing National Laboratory for Molecular Sciences, Key Laboratory of Bioorganic Chemistry and Molecular Engineering of Ministry of Education, Department of Chemical Biology, College of Chemistry and Molecular Engineering, Peking University, Beijing, 100871 China; 2 College of Life and Environmental Sciences, Minzu University of China, Beijing, 100081 China; 3 Department of Cardiology, Institute of Vascular Medicine, Department of Cardiology, Peking University Third Hospital, Beijing, 100191 China

## Abstract

G-quadruplexes in oncogene promoters provide putative targets for transcriptional regulation. The structure of a putative G-quadruplex sequence (S1: GGAGAAGGAGGAGGTGGAGGAGGAGGG) in potassium solution in the her2 promoter has been resolved mainly through nuclear magnetic resonance (NMR) spectroscopy. By application of various NMR spectra, we proved the formation of a four-layer G-quadruplex composing of two G-tetrads and two G/A-mixed planes with a four-residues loop (A3-G4-A5-A6). Further evidence from a luciferase reporter assay, Q-RT-PCR and Western blotting indicates that S1 G-quadruplex formation can repress her2 promoter activity, and a selected G-quadruplex ligand cβ can enhance the repression by down regulating her2 transcription and expression. These findings provide a G-quadruplex target and perspective implications in her2 transcriptional regulation.

## Introduction

The proto-oncogene her2 (Human epidermal growth factor receptor-2) encodes a transmembrane growth factor receptor. The her2 gene is amplified in 25 to 30 percent of human primary breast cancers^1^, and its over-expression increases breast cancer cell (MCF-7) invasiveness and tumorigenicity^2^ and induces proliferative and antiapoptotic changes of human mammary epithelial cells^3,4^, which is a characteristic of breast cancers^5^. So far, the therapeutic strategies for treatment of breast cancer caused by her2 overexpression include using antibodies such as Herceptin^®^ to block her2 protein ligand binding^6^ and chaperone Hsp90 antagonists to prevent stabilization of the active her2 conformation at the cell surface^7,8^. Alternate strategy is to suppress her2 expression by directly targeting her2 oncogene promoter. Studies have shown that triplex formation by a pyrimidine-rich oligonucleotide can inhibit her2 transcription *in vitro*^9^, and Bis-peptide nucleic acids targeting the polypurine tract of the her2 promoter can prevent her2 gene expression^10,11^.

**Figure 1:**
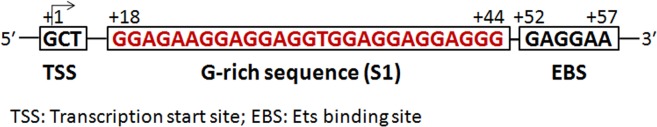
Map of the her2 promoter showing the relative positions of the S1 sequence to transcription start site (TSS) and Ets binding site (EBS).

G-quadruplex is a higher-order DNA/RNA structure that is formed by guanine-rich sequences^12–15^. Numerous studies have demonstrated that potential G-quadruplexes prevalently exist in human genomes^16–19^, and those involved in telomeres and oncogene promoters provide therapeutic targets for anti-cancer drug design^20–29^. There is a G-rich sequence (designated S1, Fig. 1) located 18 bps downstream of a major transcription initiation site on the bottom strand of the her2 promoter (NCBI, gene ID 2064). Previous studies have demonstrated that several transcription factors such as Ku70, Ku80 and PURA can bind with this G-rich sequence^30^, and a binding site for the transcription factor Ets (EBS) is located 7 nt down-stream^31^, giving potent biological relevance of this G-rich sequence.

Nuclear magnetic resonance (NMR) spectroscopy has been widely used as a powerful tool in the determination of quadruplex structures formed in human telomeres and oncogene promoters^32–36^. Previous studies by Katahira demonstrated that a GGA repeat sequence d(GGA)_8_ similar to her2 S1 can form a T:H:H:T G-quadruplex composed of two guanine tetrads (T) stacked onto two guanine-adenine heptads (H)^37,38^. Further studies showed the T:H:H:T G-quadruplex formed in the c-myb promoter can act as a target of transcriptional regulation^39,40^. In the present study, we probed the her2 G-quadruplex structure formed by an original G-rich sequence (S1) mainly through NMR spectroscopy. Our result revealed that S1 forms a similar T:H:H:T G-quadruplex composing of two G-tetrads and two G/A-mixed planes. But unlike d(GGA)_8_, there is a four-residues loop (A3-G4-A5-A6) linking the first G-tetrad and the second G/A-mixed plane in S1. Based on the structure, we found a synthetic small molecule cβ that can selectively bind to the her2 G-quadruplex with respect to its corresponding duplex DNA. Further results from luciferase assay, quantitative real-time reverse transcription polymerase chain reaction (Q-RT-PCR) and western blotting indicated that formation of the S1 G-quadruplex can repress the activity of the her2 promoter, and the G-quadruplex ligand cβ can further down-regulate her2 mRNA transcription and decrease her2 protein expression. Our work provides a G-quadruplex structure formed by the G-rich sequence in human her2 proto-oncogene promoter as well as a new strategy of her2 transcriptional regulation.

## Results and Discussion

### The her2 promoter S1 sequence formed an intramolecular parallel G-quadruplex

Circular dichroism (CD) spectroscopy is used to evaluate the formation and directions of strands of S1 G-quadruplex. Numerous studies have shown that parallel G-quadruplexes are characterized by a dominant positive CD absorption around 260 nm, whereas anti-parallel ones have a negative CD absorption at 260 nm and a positive absorption at 290 nm, and both quadruplex types show an additional characteristic positive peak at 210 nm^41–44^. The CD spectrum of S1 gives a strong positive absorption at 260 nm and another high-intensity positive band at 210 nm in 100 mM potassium solution (Fig. S1a), suggesting the formation of a parallel G-quadruplex induced by potassium ions. It is notable that unlike the CD spectra of canonical parallel G-quadruplexes which display a relatively shallow negative band at 240 nm^45^, Fig. S1a barely shows an absorption at 240 nm. This CD pattern is quite similar as that for the G-quadruplex formed by d(GGA)_8_^37^, implying a similar folding topology of S1 with d(GGA)_8_. The formation of S1 G-quadruplex is also supported by ESI mass spectrometry. The mass spectrum of S1 (Mw = 8673.7) in 100 mM ammonium solution reveals a base peak (100%) of a complex ion formed by one molecule of S1 and three ammonium ions (Fig. S1b, [S1 + 3NH_4_^+^-8H^+^]^5−^ at m/z 1742.9). As ammonium ions have a similar atomic radius to potassium ions and are located between adjacent G-tetrad planes^46,47^, the existence of the complex ion [S1 + 3NH_4_^+^-8H^+^]^5−^ indicates that S1 forms an intramolecular G-quadruplex with four planes. Furthermore, polymerase stop assays were performed to investigate the S1 G-quadruplex formation in a DNA template strand, and the results demonstrate that S1 G-quadruplex is formed and arrests Taq polymerase in a potassium-dependent manner (Supporting information, Fig. S2).

### Determination of guanines participating in G-tetrad core structure

**Figure 2:**
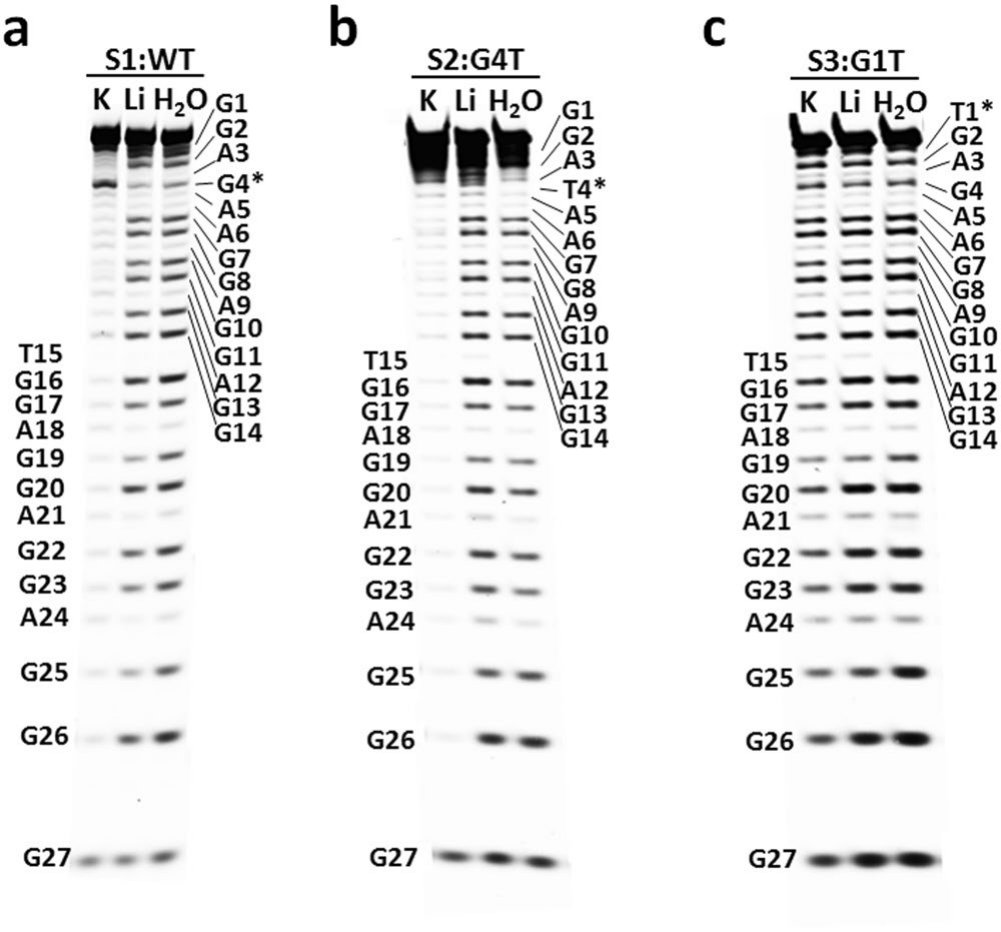
Determination of guanines participating in the G-tetrad core structure. DMS footprinting of (**a**) her2 promoter wild-type sequence S1, (**b**) the mutation sequence S2 with G4-to-T and (**c**) the mutation sequence S3 with G1-to-T. Guanines demonstrating DMS-induced cleavage are labelled on both the Left and Right. The asterisks in (**a**) mark the G4 residue which was excluded from the G-tetrad core and (**b**,**c**) the two mutation residues. The samples of S1, S2 and S3 were performed on three separate gels as shown in (**a**–**c**), respectively.

To investigate which guanines participated in the G-tetrad core structure, we performed DMS-induced strand cleavage for S1. As Fig. 2a illustrates, all S1 guanines are distinctly cleaved in pure water or 100 mM lithium solution, suggesting that all guanines are methylated and G-quadruplex is unformed under these conditions. However, in 100 mM potassium solution (30 mM Tris-HCl, pH 7.4), all other sixteen guanines show strong protection from DMS except G4 and G27, indicating that four G-tetrads are formed by these sixteen bases. Further evidence for the G-tetrad composition comes from two mutation strand cleavages. When the fourth S1 guanine (G4) was mutated to thymine (G4T, Fig. 2b), the G-quadruplex structure remains in potassium solution, and all guanines except 3′-terminal G27 are protected against DMS. When the guanine participating in the G-tetrad core structure, such as G1, was mutated to thymine (G1T, Fig. 2c), the G-quadruplex structure collapsed even in potassium solution, and all of the guanines were distinctly cleaved. This result is supported by the NMR spectra of S1–S3 in 100 mM potassium solution (30 mM Tris-HCl, pH 7.4). As shown in Fig. S3, the her2 sequence S1 (WT) and S2 (G4T) show quite similar peaks at 10.2–12.0 ppm which are characteristics of G-tetrad imino protons, while S3 (G1T) shows no detectable peaks in this region. This confirms that the S1 fourth guanine (G4) is excluded from the G-tetrad core and mutation of it barely changes the quadruplex structure, while mutation of those participating in the G-tetrad core, like G1, will destroy the quadruplex structure.

### Proton assignments through low-level site-specific fully ^15^N, ^13^C-labelling method

**Figure 3:**
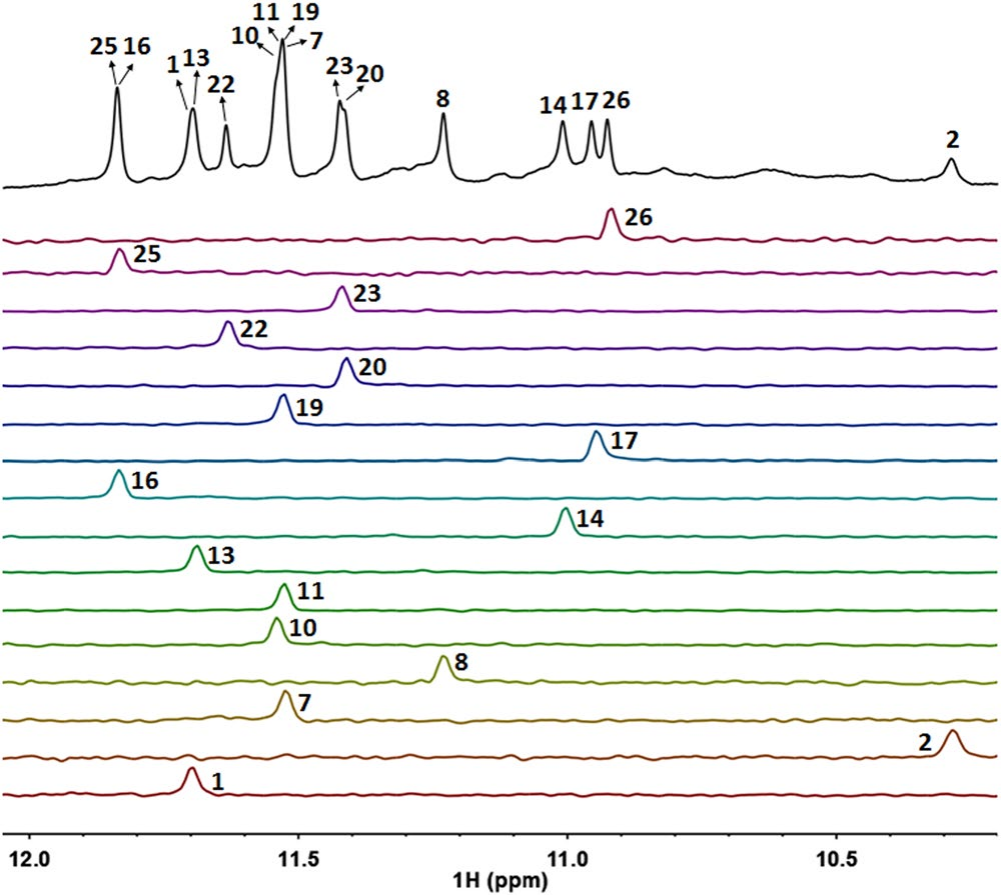
1D-^15^N-^1^H-HSQC spectra of site-specific low-enrichment (6–8%) ^15^N, ^13^C-labeled samples of S1 for imino proton assignments. Conditions: 1.0 mM DNA, 75 mM KCl, 25 mM K_2_HPO_4_-KH_2_PO_4_, pH 7.0, in 90% H_2_O, 10% D_2_O at 25 °C.

The NMR spectroscopy method was used to resolve the G-quadruplex structure that was formed by her2 S1. Proton chemical shifts were unambiguously assigned through a site-specific low-enrichment (6–8%) ^15^N, ^13^C-isotope labelling approach^48,49^. The chemical shifts of exchangeable imino and amino protons were gained through ^15^N-^1^H-HSQC spectra (Fig. 3) and those of non-exchangeable base and sugar protons were gained through ^13^C-^1^H-HSQC spectra (Supporting information, Fig. S4). The proton assignments were also confirmed by correlations in the TOCSY, NOESY and ^31^P-^1^H-HSQC spectra. Figure 3 demonstrates the guanine imino proton assignments by 1D-^15^N-^1^H-HSQC spectra. Notably, the imino proton signals of G4 and G27 were undetectable in this region, indicating that they are excluded from the G-tetrad core hoogsteen hydrogen bonds, which is consistent with the DMS footprinting results.

### Identification of two G-tetrads and two G/A-mixed planes

**Figure 4:**
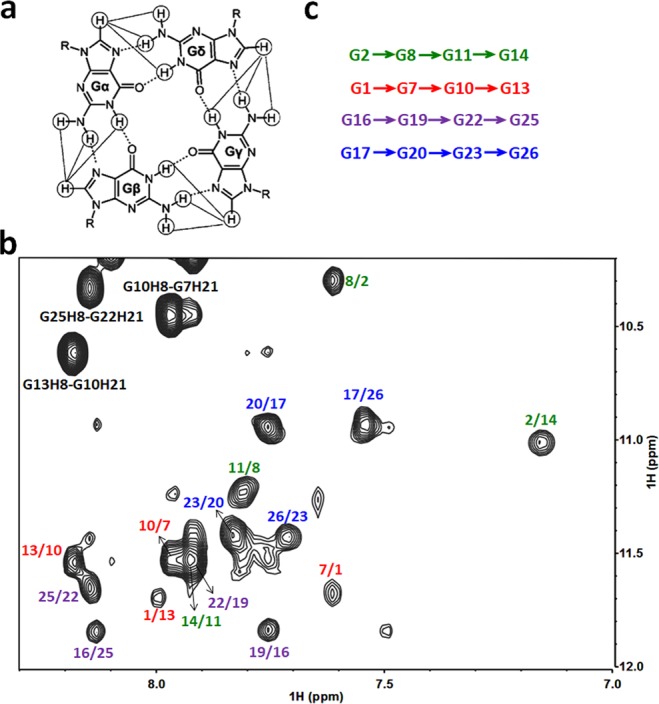
(**a**) Schematic of NOE interactions between guanosine imino/amino and H8 protons for G-tetrad determination. (**b**) The expanded H1-H8 regions of the NOESY spectrum (mixing time of 200 ms) of her2 S1. The imino H1 and aromatic H8 proton correlations within each of the four G-tetrad layers are marked in green, red, purple and blue with m/n representing GmH1-GnH8 correlation, respectively. (**c**) The four G-tetrads derived from the NOE interactions in (**b**).

**Figure 5:**
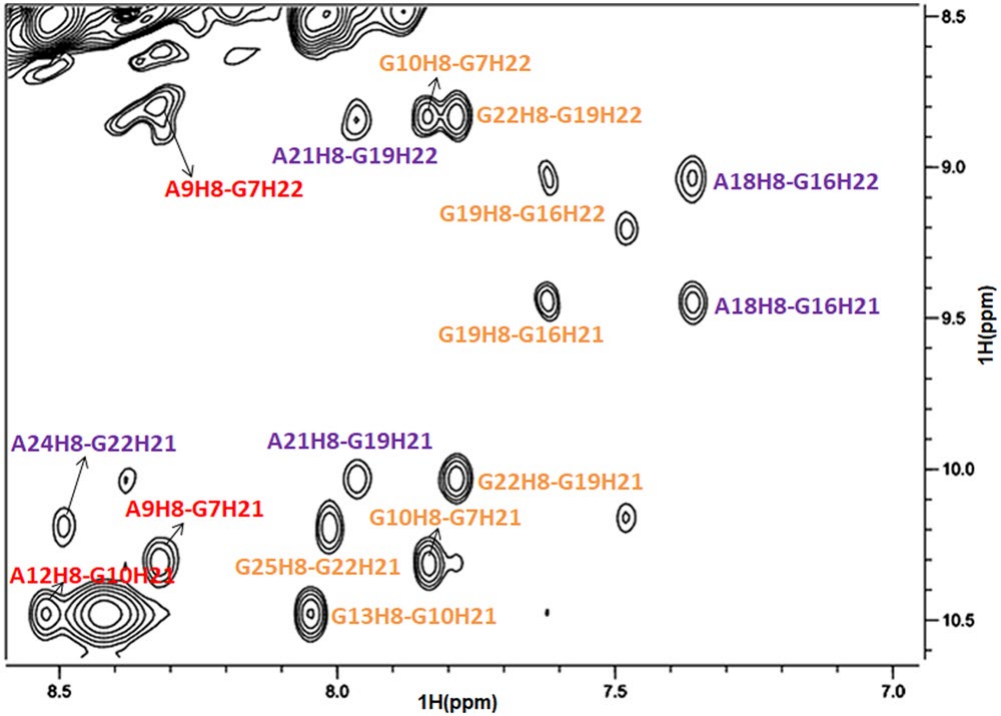
The expanded H8-H21/H22 regions of the NOESY spectrum (mixing time of 200 ms) of her2 S1. The NOE interactions within adenines and the G1•G7•G10•G13 tetrad are marked in red, and those within adenines and the G16•G19•G22•G25 tetrad are marked in purple. The brown marks show the guanine aromatic H8 and amino H21/H22 NOE interactions in G1•G7•G10•G13 and G16•G19•G22•G25 tetrads.

The characteristic NOE correlations between imino and H8 protons reveal the G-tetrad composition (Fig. 4a). Figure 4b shows the GH8-GNH1 NOE interactions from which it can be concluded that G2•G8•G11•G14, G1•G7•G10•G13, G16•G19•G22•G25 and G17•G20•G23•G26 form four tetrads of the G-quadruplex (Fig. 4c). Further evidence for the G-tetrad formation comes from the existence of NOE interactions of GH8-GNH21/22 (marked in brown in Fig. 5) in G1•G7•G10•G13 and G16•G19•G22•G25 tetrads.

Guanine and adenine can form a sheared G:A base-pair structure of which a strong GNH21/22-AH8 interaction can be observed in the NOE spectrum^37,38,50,51^. In Fig. 5, the correlations of A9H8-G7H21/22, A12H8-G10H21/22 (marked in red) and A18H8-G16H21/22, A21H8-G19H21/22, A24H8-G22H21/22 (marked in purple) reveal the formation of G7:A9, G10:A12, G16:A18, G19:A21 and G22:A24 base-pairs. An entire spectrum with the region corresponding to the sheared G:A base-pair interactions zoomed in is shown in Fig. S5. This result together with the presence of A9H8-G10H8, A12H8-G13H8, A18H8-G19H8, A21H8-G22H8 and A24H8-G25H8 NOE interactions (Supporting Information, Fig. S6) suggest that two adenines A9 and A12 are arranged along the outer edges of the G7•G10 and G10•G13 residues in the G1•G7•G10•G13 tetrad, and three adenines A18, A21 and A24 are arranged along the outer edges of the G16•G19, G19•G22 and G22•G25 residues in the G16•G19•G22•G25 tetrad, respectively.

The strong NOE interactions of aromatic H2 and sugar protons between adenines indicate the arrangement of the two G/A-mixed planes. The existence of strong NOE correlations (Supporting Information, Fig. S7) between A9 and A21 (A9H2-A21H1′/H2′′/H3′ and A21H2-A9H1′/H2′′), and A12 and A18 (A12H2-A18H1′/H2′ and A18H2-A12H2′/H2′′) demonstrate that these two adenines are stacked on each other, respectively. Notably, unlike G16(:A18)•G19(:A21)•G22(:A24)•G25 plane which contains three G/A sheared base-pairs, the absence of GNH21/22-AH8 interactions surrounding G1 suggests that there is no sheared base-pair formation between G1 and adenines in the G1•G7(:A9)•G10(:A12)•G13 plane. However, there are strong A24H2-A6H1′/H2′/H2′′ and A6H2-A24H1′ correlations in the expanded NOE spectrum, which indicate that A24 and A6 residues are spatially adjacent; thus, the A6 residue should be arranged outside the G1•G7 residues in the G1•G7(:A9)•G10(:A12)•G13 plane. In summary, from the total NOE spectra it can be derived that G2•G8•G11•G14 and G17•G20•G23•G26 form two tetrads, and A9, A12 and A18, A21, A24 are arranged along the outer edges of the other two tetrads and form sheared base-pairs with the neighbouring G residues, while A6 is arranged outside the G1•G7 residues, and thus form two G/A-mixed planes G1(A6)•G7(:A9)•G10(:A12)•G13 and G16(:A18)•G19(:A21)•G22(:A24)•G25.

### Solution structure of her2 G-quadruplex in the presence of K^+^

The structure of the her2 G-quadruplex formed by S1 was calculated using the CNS program based on NOE interactions (Supporting Information, Fig. S8). β and ε dihedral angle restrains according to ^31^P-^1^H-HSQC spectra (Supporting Information, Fig. S9), χ dihedral angle restrains according to H8/H6-H1′ (Supporting Information, Fig. S10) and γ dihedral angle restrains according to H3′–H5′/H5′′ and H4′–H5′/H5′′ correlations in NOESY were also used during the calculation. Structural statistics were listed in Table 1. Based on the 10 lowest energy structures, the her2 G-quadruplex structure is well defined with a RMSD of 0.88 Å for tetrads formed by all guanine residues except G4 and G27. The G/A-mixed planes are also well defined, as the RMSD of the tetrads and the adenine residues (A9, A12, A18, A21 and A24) is 0.89 Å. When A6 is included, the RMSD value increases to 1.20 Å. While for all the residues, the RMSD is 2.01 Å, indicating a much less-defined A3-G4-A5 loop structure.

**Table 1:** Structural statistics for the her2 S1 G-quadruplex.

NMR distance and dihedral constraints	
Distance restraints	
Total NOE	563
Intraresidue	329
Interresidue	170
Sequencial (|i − j| = 1)	101
Non-sequencial (|i − j| > 1)	69
Hydrogen bonds	64
Total dihedral angle restraints	60
β	8
γ	16
ε	24
χ	12
Planarity restraints	16
Structure statistics	
r.m.s.d. from idealized geometry	
Bond lengths (Å)	0.003 ± 0.0001
Bond angles (deg.)	0.68 ± 0.01
Impropers (deg.)	0.35 ± 0.01
NOE violations	
Number of violations greater than 0.5 Å	0 ± 0
r.m.s.d. of violations (Å)	0.05 ± 0.002
Dihedral angle violations	
Number of violations greater than 5°	0 ± 0
r.m.s.d. of violations (Å)	0.31 ± 0.03
Average pairwise r.m.s.d. of heavey atoms(Å)	
Tetrads	0.88 ± 0.15
with A9, A12, A18, A21, A24	0.89 ± 0.16
with A6, A9, A12, A18, A21, A24	1.20 ± 0.30
All residues	2.01 ± 0.72

**Figure 6:**
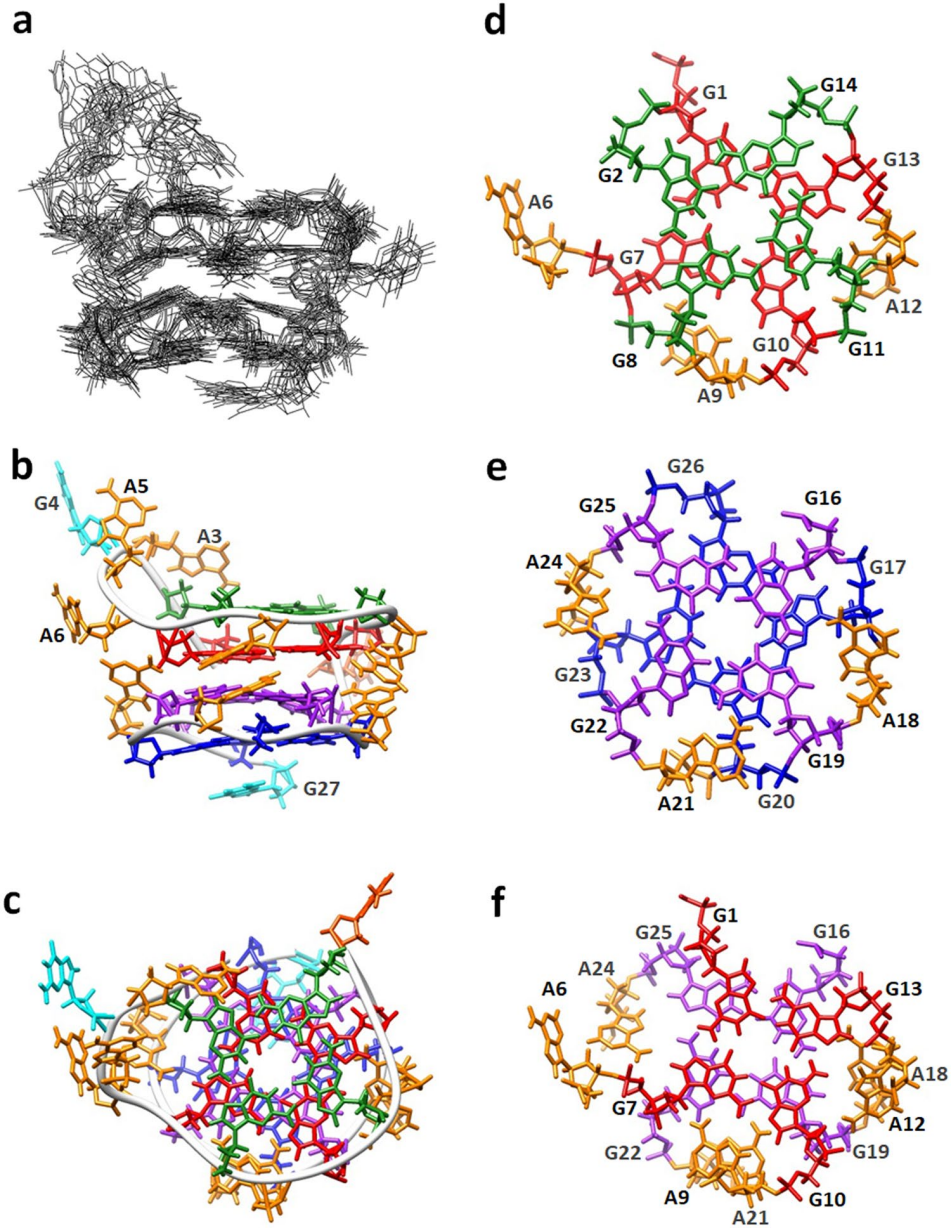
(**a**) The superimposed 10 lowest energy structures of the her2 G-quadruplex. (**b**) Side view and (**c**) top view of the average structure. The backbone is shown in Ribbon mode. Guanosines in the four planes are colored green, red, purple and blue, respectively, and those in loops are colored cyan (G4 and G27); adenosines are colored orange, and thymidine (T15) is colored orange red. Stacking interactions between (**d**) the upper tetrad (green) and G/A-mixed plane (red), (**e**) the lower G/A-mixed plane (purple) and tetrad (blue), and (**f**) the two G/A-mixed planes.

Figure 6a demonstrates the superimposed 10 lowest energy structures, and their average structure is shown in Fig. 6b (side view) and c (top view). The her2 G-quadruplex forms a four-layer structure including two outer G-tetrads (G2•G8•G11•G14 and G17•G20•G23•G26) and two inner G/A-mixed planes (G1(A6)•G7(:A9)•G10(:A12)•G13 and G16(:A18)•G19(:A21)•G22(:A24)•G25). This is also confirmed by the result that after 3 h in D_2_O solution, the imino proton signals of the guanines in the G/A-mixed planes can still be detected (Supporting information, Fig. S11) because of the strong protection of them against exchanging with D_2_O from the outer G-tetrads and surrounding adenosines. A3-G4-A5-A6 form a reversal loop linking the first and the second planes. A3 is arranged to partially stack on the G2 residue, which may cause the unusual upfield-shifted H1 (10.28 ppm) and H8 (7.17 ppm) resonance of G2. The single residue T15 links the upper and the lower half motif. The 5′-terminal G27 stacks on the outer G-tetrad.

**Figure 7:**
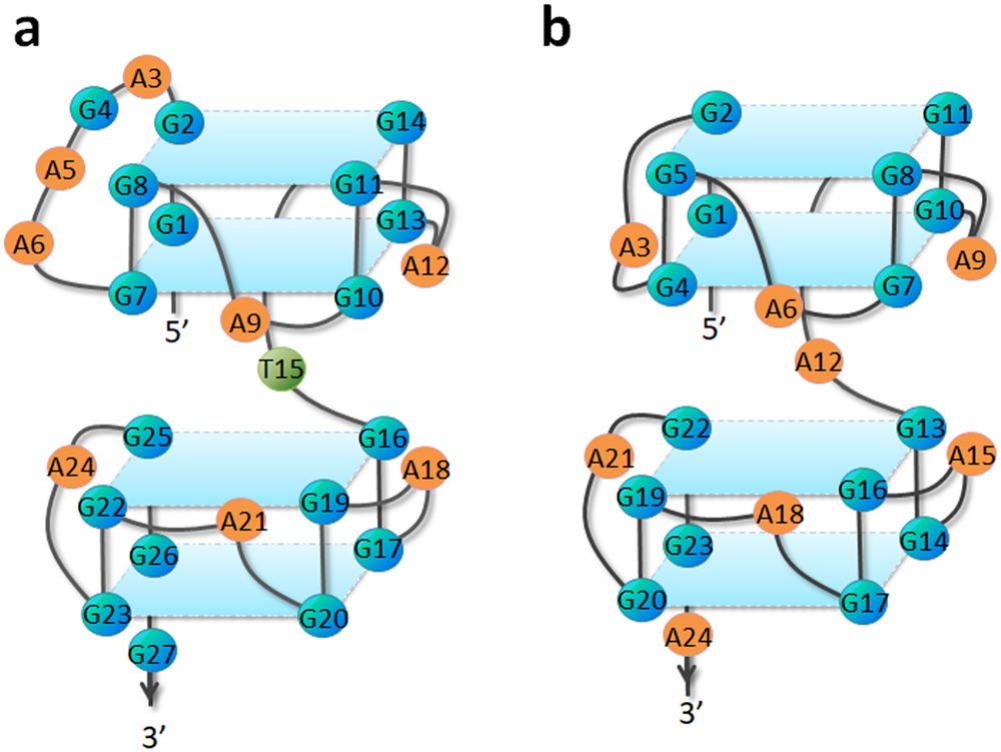
Schematic comparison of the structures of (**a**) her2 S1 and (**b**) d(GGA)_8_.

The stacking between the G-tetrads and G/A-mixed planes are shown in Fig. 6d,e,f. The hydrogen-bond directionalities of the four planes are anti-clockwise, anti-clockwise, clockwise and clockwise, respectively. It is worth noticing that the bases of A9, A12, A18, A21 and A24 are stacked on the sugar rings of G8, G11, G17, G20 and G23, respectively, which induces the extreme upfield shift of sugar H4′ of these G residues (2.63–2.80 ppm). However, because A6 is attached with A3-G4-A5 flexible linker, the co-planarity of it with the G1•G7•G10•G13 plane is poor (Fig. 7a), and thus causes a moderate H4′ up-field shift of G2 (3.13 ppm). Also, because G1 does not form sheared base-pair with A6, its amino protons are active enough that they cannot be detected at room temperature (RT), while those of the other G residues (G7, G10, G16, G19 and G22) in the G:A pair can be detected even at RT. Figure 6f demonstrates the stacking between the two G/A-mixed planes. A9 and A12 in the upper plane stacks on A21 and A18 in the lower one, respectively, which is consistent with the NOE interactions between aromatic H2 and sugar H between them. A6 is arranged on top of A24, and thus causes the A24H2-A6H1′/H2′/H2′′ and A6H2-A24H1′ correlations (Fig. S7).

The schematic structures of G-quadruplexes formed by her2 S1 sequence and d(GGA)_8_^37^ were shown in Fig. 7. In d(GGA)_8_, all of the sequential G-G-A motifs participating in the G-tetrads and G/A-mixed planes are similar with a single A residue linking the tetrad and G/A-mixed plane; while in her2 S1 G-quadruplex, except for the single A residue, there is a four-residues (A3-G4-A5-A6) loop which links the first G-tetrad and the second G/A-mixed plane. This result provides further implications of formation of G-quadruplexes with G/A-mixed planes by non-sequential GGA repeats.

### Recognition of the her2 S1 G-quadruplex by a flexible cyclic polyamide cβ

**Figure 8:**
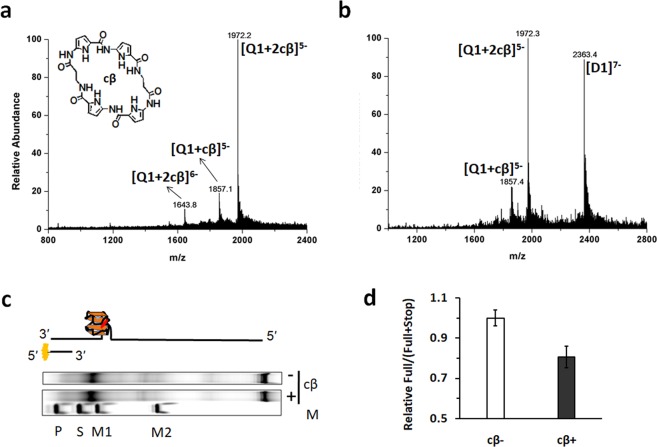
ESI mass spectra of 40 μM cβ with (**a**) 10 μM S1 G-quadruplex (marked Q1) and (**b**) 10 μM Q1 with equal doses of its corresponding duplex DNA (marked D1), which demonstrate high affinity and selectivity of cβ to Q1 with respect to D1. (**c**) Polymerase stop assay showing the primer extension band in a system with or without cβ. The marker P indicates primer, and S indicates arrested stop band. M1 and M2 represent two marker bands at 35 nt and 57 nt, respectively. (**d**) The relative intensity ratio of fully-elongated to the sum of fully-elongated and arrested bands decreased to 80% in the presence of cβ. Data represents the average of two replicates. The full-length gel is presented in Supplementary Fig. S14.

As a novel G-quadruplex structure formed by the original G-rich sequence in the her2 promoter, S1 G-quadruplex recognition implies potential importance in the regulation of her2 expression. Here, the ESI-MS method was used to evaluate the binding affinities of a series of cyclic polyamide (cα, cβ, cγ, cPT and cPTN, Supporting Information, Fig. S12) to the her2 S1 G-quadruplex. Figure 8a shows the ESI mass spectrum of a mixture solution of S1 G-quadruplex and cβ in which the base peak is a complex ion composed of one S1 G-quadruplex ion (abbreviated to Q1) and two cβ molecules ([Q1 + 2cβ]^5−^, m/z = 1972.2), and the free Q1 ion cannot be detected. While the ESI mass spectra of the mixtures of the other small molecules and the G-quadruplex (Q1) reveal no complex ions but free Q1 (for cα, cγ and cPTN, Supporting Information, Figs S13a,b,d) or an intensity of complex ion of less than 20% (for cPT, Supporting Information, Fig. S13c). These results indicate that out of the five cyclic polyamides, only cβ has high binding affinity to the her2 S1 G-quadruplex. Because the selectivity between the G-quadruplex and duplex DNA is also an important aspect for a G-quadruplex ligand, we generated a mixture of G-quadruplex and its corresponding duplex DNA by mixing S1 and its complementary cytosine-rich strand C1 (Mw = 7890.3) at a molar ratio of 2:1. When four cβ equivalents were added to the mixture, the ESI mass spectrum (Fig. 8b) revealed a base peak of a complex Q1 ion and two cβ molecules ([Q1 + 2cβ]^5-^), while no complex duplex DNA ion ([S1 + C1–7H^+^]^7-^, m/z = 2363.4, abbreviated to [D1]^7-^) and cβ were observed, indicating that cβ selectively recognized the her2 G-quadruplex over the duplex DNA. Further evidence from a polymerase stop assay supported this result. When cβ was added to the system, the ratio of the fully-elongated band to the sum of arrested and fully-elongated band of primer extension using the template containing S1 G-quadruplex decreased to 80% (Fig. 8c,d), demonstrating that cβ bound to the her2 S1 G-quadruplex and inhibited the sliding of Taq polymerase along the template.

### Regulation of her2 transcription by the G-quadruplex ligand cβ

**Figure 9:**
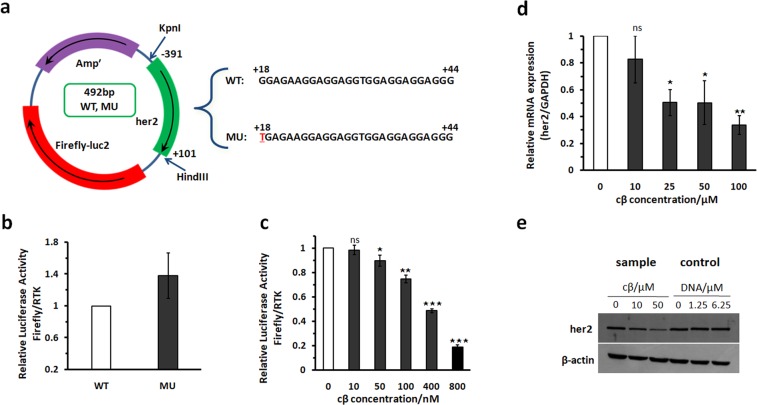
(**a**) Schematic representation of the constructed WT and MU plasmids. Relative luciferase activity of (**b**) the two constructed plasmids and (**c**) the WT plasmid treated with 0–800 nM cβ. The dose-dependent (**d**) Q-RT-PCR assay and (**e**) Western blotting demonstrating that cβ inhibited her2 gene transcription and protein expression. *P < 0.1, **P < 0.01, ***P < 0.001, ns indicates no significance.

To investigate the function of the selected G-quadruplex ligand cβ in regulation of her2 transcription, we performed a luciferase reporter assay experiment according to the protocols described previously^39,52^. First, we evaluated the function of the S1 G-quadruplex in the her2 promoter by constructing two plasmids including wildtype (WT) and a mutant (MU) sequence of S1 (Fig. 9a). Based on our previous study, the WT sequence formed an S1 G-quadruplex structure, and the MU sequence destroys the G-quadruplex. As illustrated in Fig. 9b, the relative luciferase activity of the MU plasmid increased by nearly 40% compared with that of the WT plasmid, indicating that the formation of the S1 G-quadruplex can inhibit her2 promoter activity, while its destruction (MU) up-regulates the activity. We then evaluated the influence of cβ on her2 transcription by adding 0–800 nm cβ to the luciferase reporter assay system. As illustrated in Fig. 9c, relative luciferase activity of the her2 plasmid decreased gradually to less than 20% when the cβ concentration increased to 800 nM, indicating inhibition on her2 promoter activity by cβ. This result was supported by further RT-PCR and Western blotting experiments. The relative expression level of her2 mRNA gradually decreased to nearly 30% after MCF-7 cells were treated with 0–100 μM cβ (Fig. 9d), demonstrating that cβ can inhibit her2 transcription. Western blotting experiments indicated that the relative her2 protein expression level reduced to 70% or 20% when 10 or 50 μM cβ along with S1 DNA as a carrier was added to cells (sample in Fig. 9e), while carrier DNA without cβ had no influence on her2 expression (control in Fig. 9e). In summary, our study demonstrates a synthetic small molecule cβ, which can selectively bind to the her2 promoter S1 G-quadruplex and inhibit her2 proto-oncogene transcription and expression.

## Conclusion

To conclude, our work revealed a G-quadruplex structure formed by an original G-rich sequence (S1: GGAGAAGGAGGAGGTGGAGGAGGAGGG) in the her2 proto-oncogene promoter. The A3-G4-A5-A6 loop in the S1 G-quadruplex provides further evidence of four-layer G-quadruplex formation with G/A-mixed planes by non-sequential GGA repeats. We also demonstrated that the S1 G-quadruplex can inhibit her2 promoter activity, and a selected S1 G-quadruplex ligand cβ can suppress her2 transcription and expression. Since the elevated expression of her2 has been shown to be strongly associated with the development and progression of aggressive breast cancers, our study provides a putative G-quadruplex target for breast cancer treatment. Further efforts may be made on the study of the her2 S1 G-quadruplex ligands, either bind to the tetrad core or the loops, to stabilize the G-quadruplex and hence repress the her2 oncogene expression for related pathologies regulation.

## Material and Methods

### Sample preparation

Unlabeled oligonucleotides were purchased from Sangon Biotech. (Beijing, China). Site-specific labelled (^15^N and ^13^C) oligonucleotides were synthesized by Takara Biotech. (Dalian, China) using nucleotides labelled with ^15^N and ^13^C from Cambridge Isotope Laboratories, Inc. (MA, USA). Small molecules (cα, cβ, cγ, cPT and cPTN) were synthesized in our lab^53,54^. DNA samples for NMR spectroscopy were purified by successive dialysis against 0.5 M KCl solution and then against water^55–58^. For a stable G-quadruplex solution generation, purified S1 was diluted in K^+^ buffer containing 75 mM KCl, 25 mM K_2_HPO_4_-KH_2_PO_4_ (pH 7.0) at a final concentration of 1.0 mM strands for NMR spectra scanning.

### NMR Spectroscopy

NMR spectra were recorded on Bruker Advance 800 MHz, and Varian VNMRS 600 MHz spectrometers equipped with cryogenic probes at 25 °C^57,59–64^. Resonances were assigned unambiguously by 1D-^15^N-^1^H-HSQC and 1D-^13^C-^1^H-HSQC experiments using a site-specific low-enrichment (6–8%) fully ^15^N, ^13^C-labeling approach^48,49^. Assignments were confirmed through standard 2D NMR experiments including TOCSY (τ_m_ of 120 ms) and NOESY (τ_m_ of 50, 200 and 250 ms). Spectra in water solutions were performed with water suppression using watergate W5 pulse sequence with gradients. Relaxation delays were set to 2 s. The acquired sizes were set to 1024 × 320, and the final spectral sizes were 1024 × 1024. 2,2-dimethylsilapentane-5-sulfonic acid (DSS) was used as an internal standard for the ^1^H chemical shifts assignments.

NMR spectra were processed and analyzed using MestReNova software (http://mestrelab.com/). Cross-peaks were assigned and integrated using MestReNova peak fitting function and volume integration. The inter-proton distance restraints were obtained from a 2D NOESY spectrum with a mixing time of 200 ms using the average volume integral of the H1′-H2′′ cross-peaks (2.20 Å) as a reference^34,35,63^. A certain value of bound was added to each of the calculated distance, that is 0.8 Å added for the calculated distance of 0.0–2.7 Å, 1.5 Å added for 2.7–3.2 Å, 2.0 Å added for 3.2–4.0 Å, and 2.5 Å added for 4.0–6.0 Å. Further 1.0 Å was added for the imino H1 and aromatic H8 pairs in the G-tetrad core and 1.8 Å was used as a lower bound distance for all proton pairs^37,38^. Atoms participating in experimentally identified G-tetrads were restrained with distances corresponding to ideal hydrogen bond geometry^57–60^. Dihedral angle restraints for the β and ε torsion angles were derived from ^31^P-^1^H-HSQC spectra with coupling constants of 5 to 25 Hz as described previously^37,38^. Thus, the β torsion angles of G2, A3, A6, G8, G11, G17, G20 and G23 were constrained to 180 ± 20°, and the ε torsion angles of all residues except G2, A6 and G27 were constrained to −120 ± 45°. The γ torsion angles of A3, G4, A5, A6, G7, G10, G13, G14, G15, G16, G18, G19, G22, G23, G25 and G27 were constrained to 60 ± 20° derived from the relative intensities of H3′–H5′/H5′′ and H4′–H5′/H5′′, and χ torsion angles of the anti-residues G2, A5, A6, A9, G10, G13, G17, A18, G20, A21, A24 and G25 were constrained to 220 ± 40° derived from the relative intensities of H8/H6-H1′ as described previously^56,57,60^.

The NMR structure of the her2 promoter S1 sequence was calculated using the Simulated Annealing protocol of the Crystallography & NMR system (CNS) version 1.3 program^65,66^. Briefly, an initial random conformation of S1 sequence is generated and imposed to simulated annealing using experimentally measured interproton distance estimates, hydrogen bonds and dihedral angle restraints. In total, 100 trial structures were calculated with 100 cycles of final minimization, and 74 structures were accepted based on violations. A planarity restraint of 10 kcal•mole^−1^•Å^−2^ was imposed on the G-tetrads during the annealing. Ten final structures were selected out of the 74 accepted ones based on their minimal energy terms and number of NOE violations. The structures were viewed using the UCSF Chimera program^67^.

### ESI Mass Spectrometry (ESI-MS)

ESI-MS experiments were performed using the Finnigan LCQ Deca XP Plus ion-trap mass spectrometer (CA, USA). S1 (10 μM) was dissolved in 100 mM NH_4_OAc solution (pH 6.9) and pre-incubated at 37 °C for 8 h to generate the G-quadruplex. Each of the synthetic small molecule ligands were dissolved in 1:9 (v/v) DMSO/CH3OH solution to a stock concentration of 500 μM and added to the DNA samples for binding affinity investigation. Duplex DNA were generated by mixing 10 μM S1 and the equivalent of its complementary cytosine-rich strand (designated C1). For each sample, 25% (v/v) methanol was added to improve electrospray efficiency. Negative ion mode was used with a spray voltage of 2.7 kv and a capillary temperature of 140 °C. The infusion rate was 2 μl/min, and scans lasting 5 min were summed for each spectrum.

### Circular Dichroism (CD) Spectroscopy

CD experiments were performed with a JASCO J-815 CD spectrometer (Tokyo, Japan). S1 (10 μM) was dissolved in 30 mM Tris-HCl (pH 7.4) buffer with 100 mM or without KCl. Samples were pre-annealed at 90 °C for 10 min and then slowly cooled down to 25 °C (more than 8 h) before being tested in a 0.1 cm path-length cuvette. The scanning wavelength range was from 200 to 400 nm, and three times scans were averaged for each spectrum.

### Dimethyl Sulfate (DMS) Footprinting

DMS footprinting experiments were performed as follows^39,52,68^. 3′-6-FAM labelled oligonucleotides (S1–S3, Supporting Information Table S1) were diluted to 0.1 μM in 100 μl 30 mM Tris-HCl (pH 7.4) in the presence of H_2_O, 100 mM LiCl or KCl. DNA samples were then annealed at 90 °C for 10 min and slowly cooled down to 4 °C (more than 8 h) before being treated with 10% (v/v) DMS for 1.5 min at 25 °C. The reaction was quenched by adding 100 μl stop solution (20 μl β-mercaptoethanol, 40 μl 3 M sodium acetate, 50 μg sperm DNA). The DMS-treated DNA samples were extracted with a Tris-phenol-chloroform solution (pH 8.0) and then precipitated using ethanol at −80 °C before being cleaved by 10% (v/v) piperidine at 90 °C for 30 min. The cleaved DNA samples were separated on a 20% sequencing gel at 1500 v for 4 h in a 4 °C cold room and imaged using a GE Healthcare Typhoon9400 gel scanner (CT, USA).

### Polymerase Stop Assays

Polymerase stop assays were performed as follows^39,52,69^. Briefly, the her2 promoter S1 sequence was inserted into a general template and annealed with a 5′-6-FAM labelled primer (sequences listed in supporting information Table S1) in a PCR buffer with 0–150 mM KCl at 95 °C for 5 min before being slowly cooled down to 25 °C over 8 h. The general template without S1 sequence was used as a control. To verify the binding properties of the small molecule ligand, 0 or 10 doses of cβ were added to the template-primer system in the presence of 150 mM KCl. Afterward, Taq polymerase (BBI, MA, USA) was added to conduct primer extension at 37 °C for 15 min. The reaction was quenched on ice, and products were separated on a 12% denaturing gel before imaging using a GE Healthcare Typhoon9400 gel scanner (CT, USA).

### Luciferase Assay

The luciferase assay experiments were performed as follows^39,52^. An approximately 500 bp sized fragment of the human her2 promoter (from −414 to +78) including the S1 sequence was generated via PCR from Hela cells and inserted into a pGL4.10-basic vector (Promega, WI, USA) for construction of the wild-type (WT) plasmid. The mutant plasmid (MU) containing S3 instead of S1 was also constructed as described, and both plasmids were verified via automated sequencing.

The cultured MCF-7 cells were plated at a density of 1 × 10^5^ cells/well into a 24-well plate and incubated at 37 °C overnight. To verify the influence of the mutation on promoter activity, 400 ng WT or MU plasmids were transfected into MCF-7 cells using Lipofectamine 2000 (Invitrogen, CA, USA) as a transfection reagent. p-RL-TK vector (40 ng) was co-transfected as an internal control. To verify the effect of the small molecule ligand on her2 promoter activity, 0–800 nM cβ was mixed with the WT plasmid and transfected into MCF-7 cells. Luciferase activities were measured using a dual luciferase reporter assay system (Promega, WI, USA) according to the manufacturer′s protocol. Relative luciferase activities were acquired by normalizing the ratio of firefly luciferase activity to renilla luciferase activity of the mutant construct with that of the wild-type construct.

### Q-RT-PCR

Q-RT-PCR experiments were performed as described previously^52^. Cultured MCF-7 cells were split into a 6-well plate at a density of 8 × 10^5^ cells/well and incubated at 37 °C overnight. Powdered cβ was dissolved in DMSO to generate a 10 mM stock solution. A mixture of this stock solution and PEI (Mw = 25000, linear) at a mass ratio of 1:0.8 was generated and diluted with complete growth medium to form a working solution (0–100 μM), which was used to treat MCF-7 cells at 37 °C overnight. Cells were harvested, and total RNA was extracted using Trireagent (Invitrogen, CA, USA) according to the manufacturer′s instructions. TransScript II All-in-One-First-Strand cDNA SuperMix kits (TransGen, Beijing, China) were used to generate cDNA. Forward and reverse primers (Supporting information, Table S1) were added to the cDNA solution for the real-time PCR reaction using SYBR Green PCR Master Mix (Applied Biosystems, CA, USA), and GAPDH was used as a control. The reaction was performed in an Eppendorf Realplex Real-time PCR system (Hamburg, Germany).

### Western Blotting

Western blotting experiments were performed as described previously^52^. Cultured Hela cells were split into a 6-well plate (6 × 10^5^ cells/well) and incubated at 37 °C overnight. Powdered cβ was dissolved in DMSO to generate a 10 mM stock solution and mixed with her2 S1 DNA solution (0.5 mM) at a volume ratio of 4:1 to generate a cβ-DNA mixture before annealing at 90 °C. A serial volume of this cβ-DNA mixture was diluted using complete growth medium to a working concentration of 0–50 μM for cβ. Lipofectamine 2000 (Invitrogen, CA, USA) was used as a transfection reagent, and DMSO was the control. The samples were harvested using a general procedure and then loaded on a 10% SDS gel and transferred to a nitrocellulose membrane at 45 v for 18 h in a 4 °C cold room. The samples were then blocked with 5% non-fat milk at room temperature for 1 h and probed with specific primary antibodies at 4 °C overnight. Target proteins were visualized via treatment with HRP-linked secondary antibodies.

## Supplementary information

Supporting Information
