# Supplementary material for: Exploration of the Structure and Recognition of a G-quadruplex in the her2 Proto-oncogene Promoter and Its Transcriptional Regulation
Source: Sci Rep. 2019 Mar 8;9:3966. doi: 10.1038/s41598-019-39941-5 (PMC6408435; doi:10.1038/s41598-019-39941-5)

**Exploration of the Structure and Recognition of a G-quadruplex in the her2 Proto-oncogene Promoter and Its Transcriptional Regulation**

Xiaojie Cui^1,2^*, Han Chen^1^, Qiang Zhang^1^, Ming Xu^3^, Gu Yuan^1^ and Jiang Zhou^1^*

**Supplementary information**

**Table S1. Sequences used in this study.**

| Experiments | Codes | Sequences (5′-3′) |
| --- | --- | --- |
| DMS foot-printing | S1 | GGAGAAGGAGGAGGTGGAGGAGGAGGG |
|  | S2 | GGATAAGGAGGAGGTGGAGGAGGAGGG |
|  | S3 | TGAGAAGGAGGAGGTGGAGGAGGAGGG |
| Polymerase stop assay | template | TCCAACTATGTATACGGAGAAGGAGGAGGTGGAGGAGGAGGGTTAGCGGCACGCAATTGCTATAGTGAGTCGTATTA |
|  | templatecontrol | TCCAACTATGTATACTTAGCGGCACGCAATTGCTATAGTGAGTCGTATTA |
|  | primer (P) | TAATACGACTCACTATAGCAATTGCGTG |
|  | stop (S) | TAATACGACTCACTATAGCAATTGCGTGCCGCTAA |
| Q-RT-PCR | forward | CTCTGAGACTGATGGCTACGTTGC |
|  | reverse | CGTCTTTGACGACCCCATTCTTCC |

**Figure S1.** (a) CD spectrum of 10 μM S1 in 30 mM Tris-HCl (pH 7.4) with 100 mM KCl. The absorption peak at 260 nm indicates that S1 forms a parallel G-quadruplex. (b) ESI mass spectrum of 10 μM S1 in 100 mM NH_4_OAc, 25% CH_3_OH (pH 6.9). The complex ions with three ammoniums demonstrate four planes in the G-quadruplex.

**Figure S2.** Polymerase stop assays. (a) the primer extension band in a system containing 0-150 mM KCl. As the concentration of KCl increases, the intensity of fully-elongated band decreases and that of arrested band increases. The marker P indicates primer, and S indicates stop band. (b) potassium dependence of the ratio of fully-elongated band intensity to the sum of fully-elongated and arrested bands intensity. (c) the primer extension band using the general template without S1 as a control. The intensity of fully-elongated band remains constant under 0-150 mM KCl conditions, which excludes the influence of KCl concentration on polymerase activity.

**Figure S3.** ^1^H NMR spectra at 10.2-12.0 ppm of (a) her2 promoter wild-type sequence S1 and (b) the mutation sequence S2 with G4-to-T and (c) the mutation sequence S3 with G1-to-T. Conditions: 1.0 mM DNA, 100 mM KCl, 30 mM Tris-HCl, pH 7.4, in 90% H_2_O, 10% D_2_O at 25°C.

**Figure S4.** 1D-^13^C-^1^H-HSQC spectra of site-specific low-enrichment (6-8%) ^15^N, ^13^C-labeled samples of S1 for aromatic and sugar proton assignments. Conditions: 1.0 mM DNA, 75 mM KCl, 25 mM K_2_HPO4-KH_2_PO_4_, pH 7.0, in 90% H_2_O, 10% D_2_O at 25℃.

**Figure S5.** (a) The entire 2D-^1^H-^1^H NOESY spectrum (mixing time of 200 ms) of S1. Red dot square illustrates the region corresponding to the sheared G:A base-pair interactions. (b) zoomed in spectrum of (a). The NOE interactions within adenines and the G1•G7•G10•G13 tetrad are marked in red, and those within adenines and the G16•G19•G22•G25 tetrad are marked in purple. The brown marks show the guanine aromatic H8 and amino H21/H22 NOE interactions in G1•G7•G10•G13 and G16•G19•G22•G25 tetrads. Conditions: 1.0 mM DNA, 100 mM KCl, 30 mM Tris-HCl, pH 7.4, in 90% H_2_O, 10% D_2_O at 25°C.

**Figure S6.** The expanded H8-H8 regions of the NOESY spectrum (mixing time of 200 ms) of her2 S1. The H8-H8 interactions between adenines and guanines within the G1•G7•G10•G13 tetrad (G10H8-A9H8 and G13H8-A12H8) are marked in red, and those between adenines and guanines within the G16•G19•G22•G25 tetrad (G19H8-A18H8, G22H8-A21H8 and G25H8-A24H8) are marked in purple.

**Figure S7.** The expanded adenine aromatic H and sugar H correlation regions of the NOE spectrum (mixing time of 200 ms) of her2 S1. The NOE interactions between (a) H2 and H2′/H2′′ and (b) H2 and H1′/H3′ of adenines in two G/A-mixed planes are marked in red. Cross-peaks a-m are as follows: a, A12H8-A12H2′; b, A12H8-A12H2′′; c, A24H8-A24H2′; d, A9H2-G8H4′; e, A9H2-G8H5′; f, A3H8-A3H2′′; g, A12H8-A12H3′; h, A12H8-A12H1′; i, A12H8-G11H1′; j, A24H8-A24H3′; k, A24H8-A24H1′; l, A9H8-A9H3′; m, A3H8-A3H1′.

**Figure S8.** Schematic diagram of NOE interactions between G/A and A residues. Guanosines are boxed in white and adenosines are boxed in gray. The marked NOE connectivities define the conformation of the loop and the interaction between the two G/A-mixed planes.

**Figure S9.** ^31^P-^1^H-HSQC spectrum (coupling constant *J*=10 Hz) of her2 S1 for the β and ε dihedral angle restraints. The NnH3′ and N(n+1)H4′/H5′/H5′′ represents N(n+1)P-NnH3′ and N(n+1)P-N(n+1)H4′/H5′/H5′′ correlations, respectively. The ^31^P chemical shifts are referenced to H_3_PO_4_. For G2, A3, A6, G8, G11, G17, G20 and G23, the H5′/H5′′-P peaks are clearly absent and thus the β torsion angles of them were constrained to 180±20°; For all residues except G2, A6 and G27, the H3′-P peaks are clearly observed and thus the ε torsion angles of them were constrained to -120±45°.

**Figure S10.** The expanded H8/H6-H1′ regions of the NOESY spectrum (mixing time of 200 ms) for the χ dihedral angle restraints. The intra residue H8/H6-H1′ NOE correlations are labeled with residue numbers. The asterisks represent interactions which can be seen at lower spectral threshold. For G2, A5, A6, A9, G10, G13, G17, A18, G20, A21, A24 and G25, the weak H8 -H1′ correlations indicate anti-glycosidic bonds and thus their χ angles were constrained to 220±40°.

**Figure S11.** NMR spectrum of imino protons of her2 S1 after 3 h in D_2_O solution. Proton assignments are listed over the peaks.

**Figure S12.** The structures of cyclic polyamide small molecules used in the her2 S1 G-quadruplex ligand selection.

**Figure S13.** ESI mass spectra of 40 μM (a) cα, (b) cγ, (c) cPT and (d) cPTN with 10 μM S1 G-quadruplex (marked Q1), which demonstrate poor binding affinity of these small molecules to Q1.

**Figure S14.** The full-length gel image showing the arrest of polymerase by her2 G-quadruplex in the absence or presence of cβ (Band 2 and 4, respectively). The wide-spectrum G-quadruplex ligand chelerythrine (Band 3) was used as a positive control. Two replicates (1 and 2) were made to get an average data as shown in Figure 8d.

Fig S1.


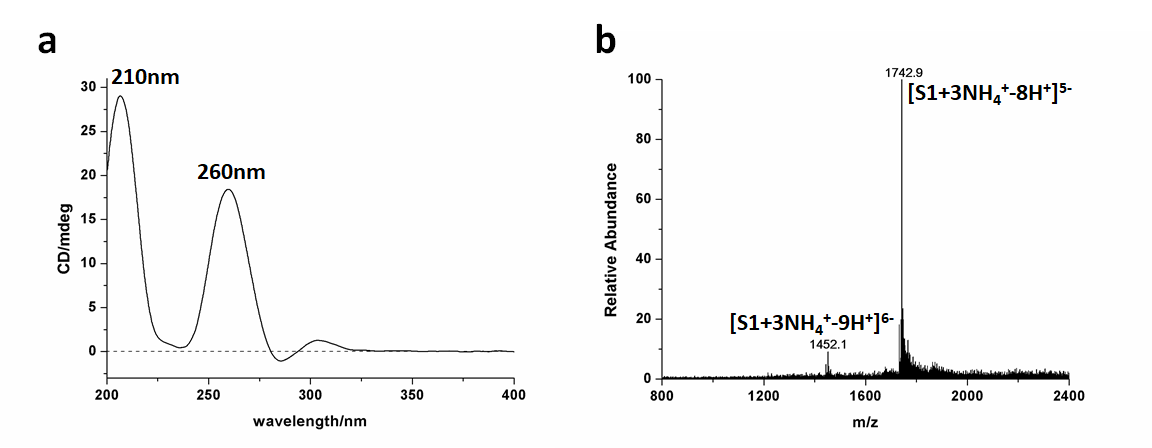


Fig S2.


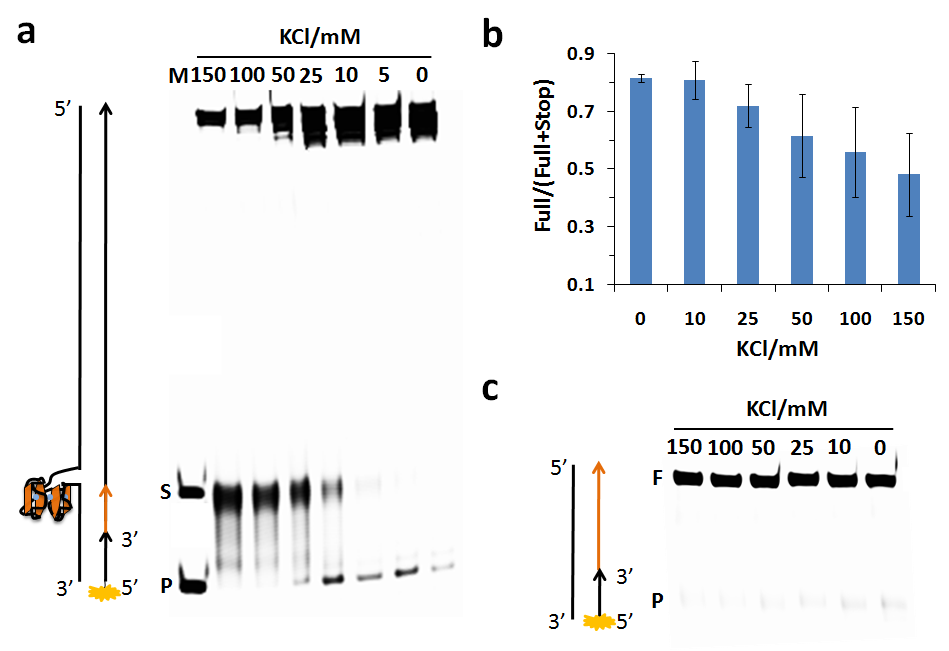


Fig S3.


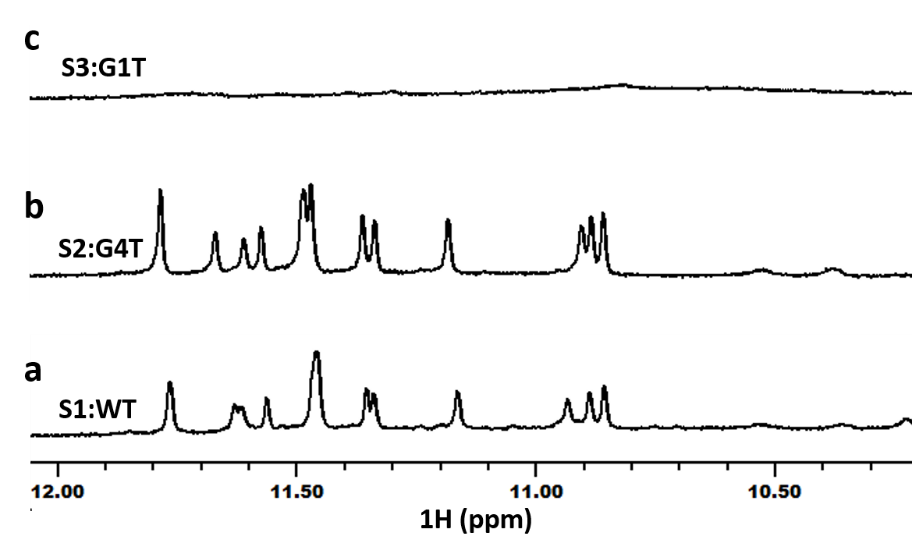


Fig S4.


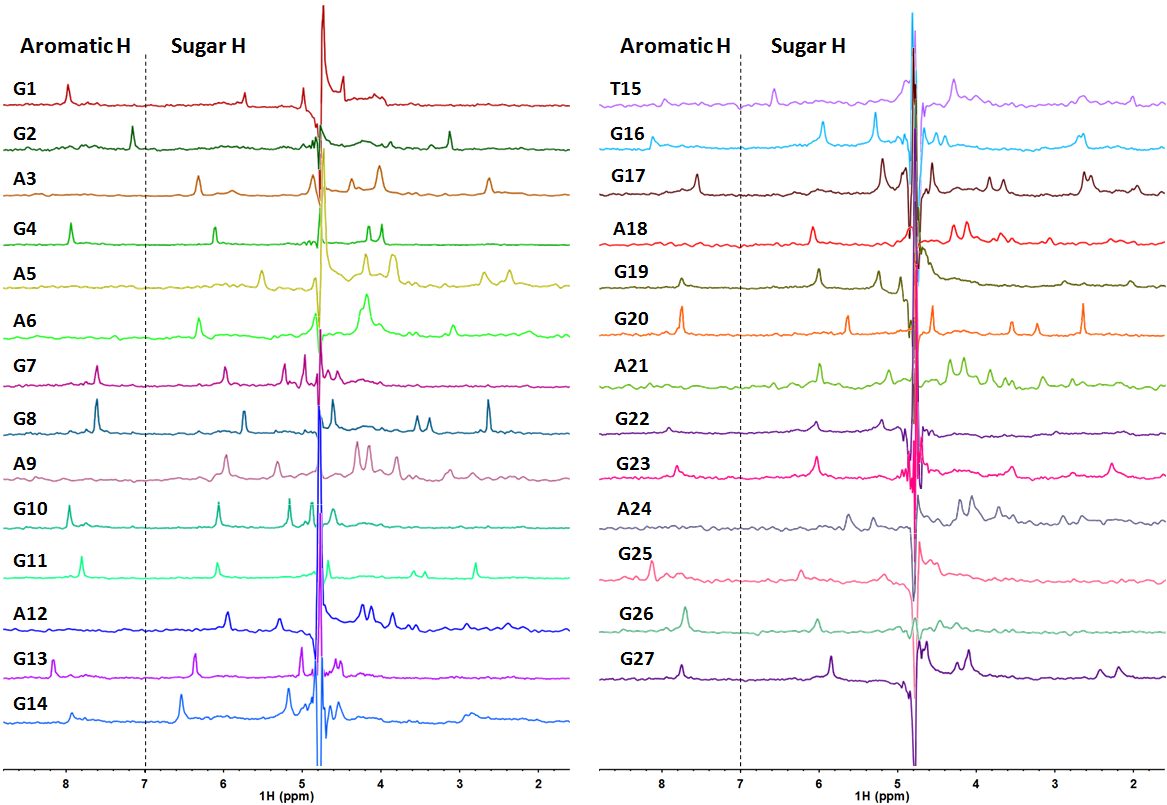


Fig S5.


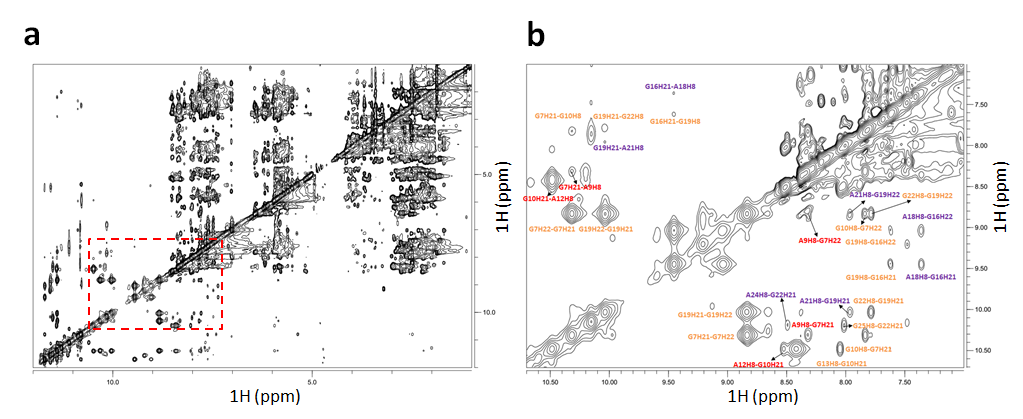


Fig S6.


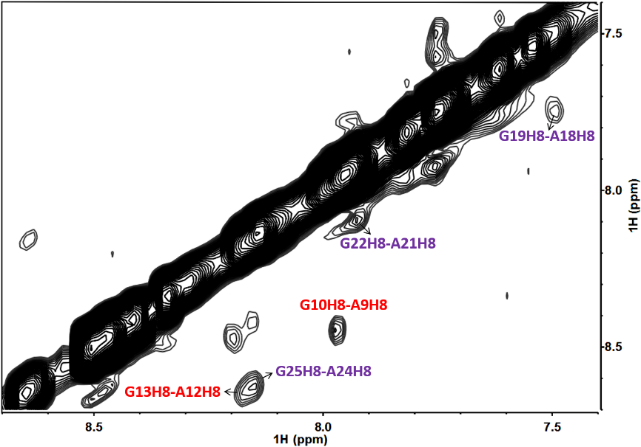


Fig S7.


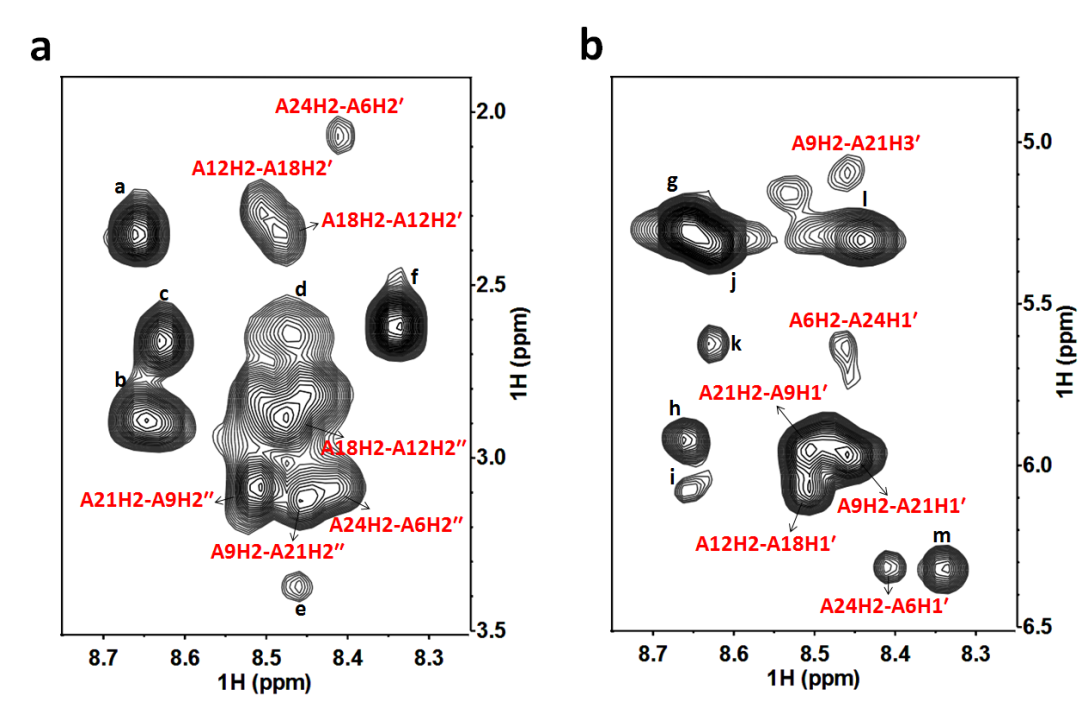


Fig S8.


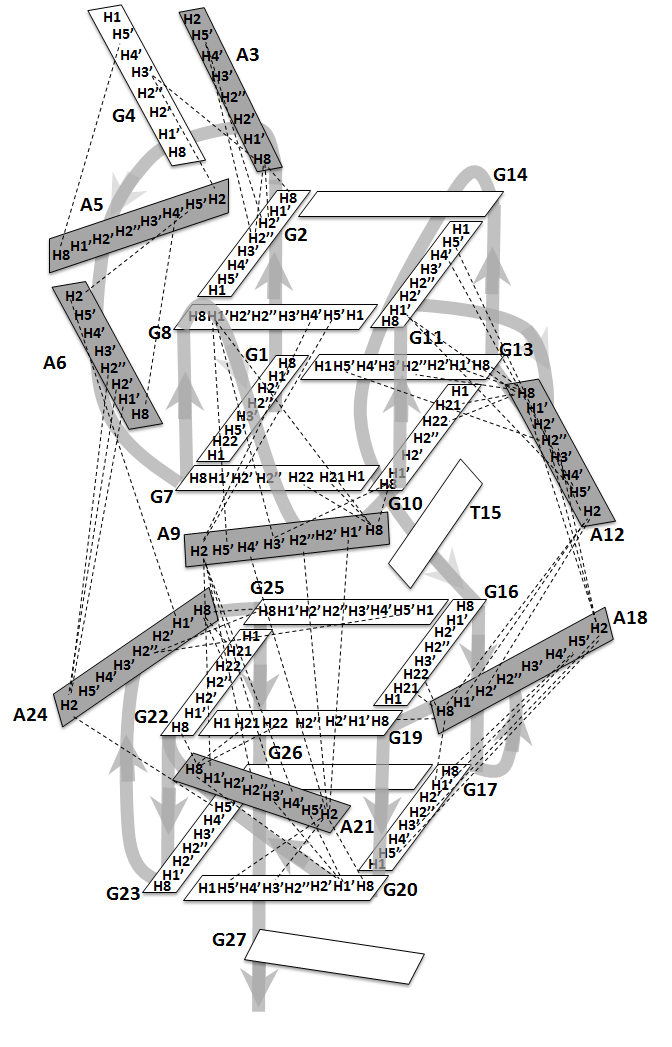


Fig S9.


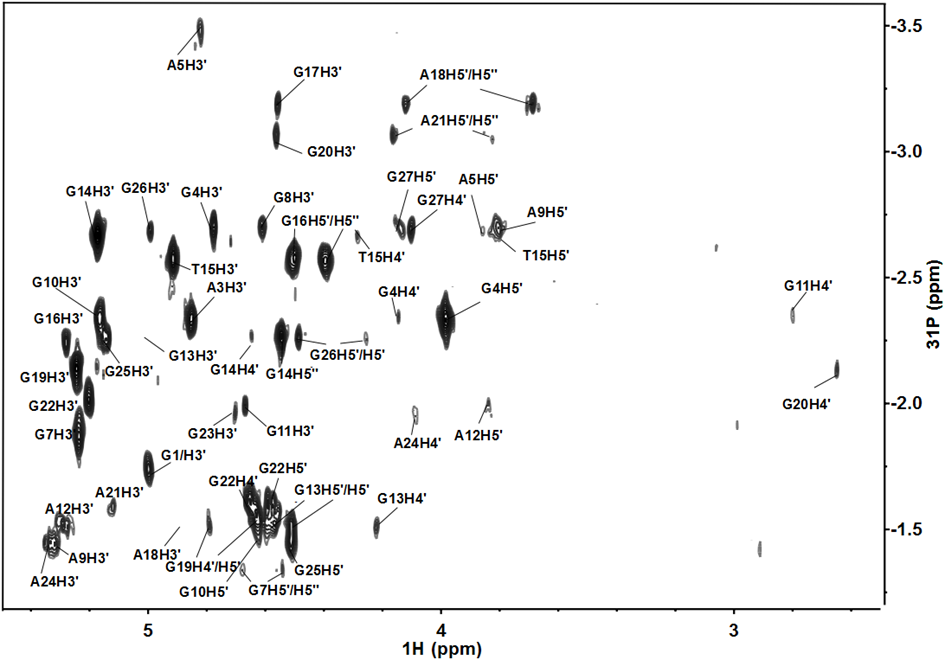


Fig S10.


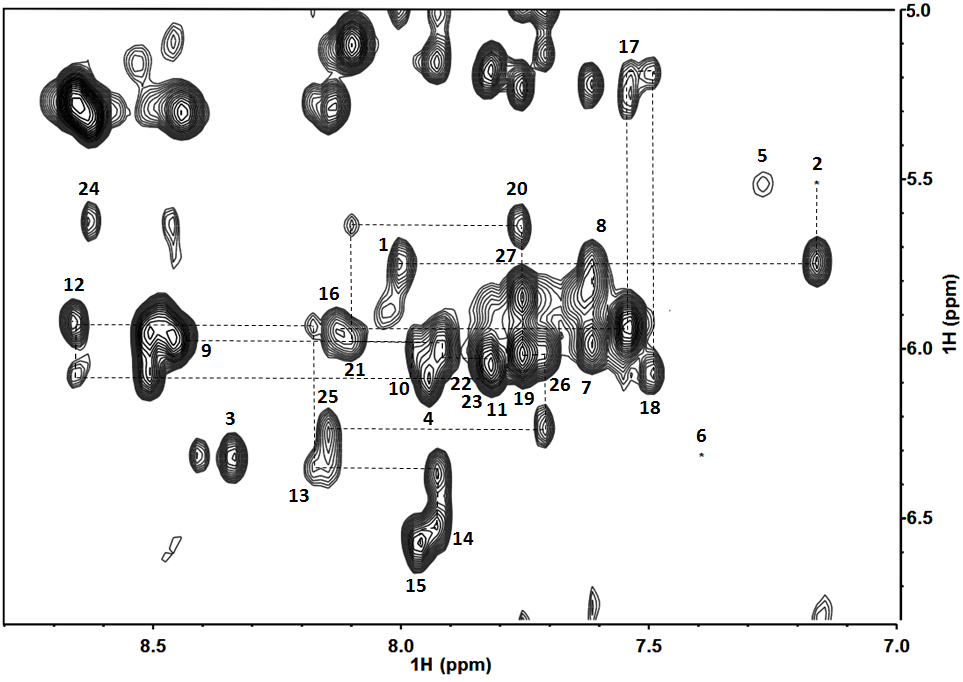


Fig S11.


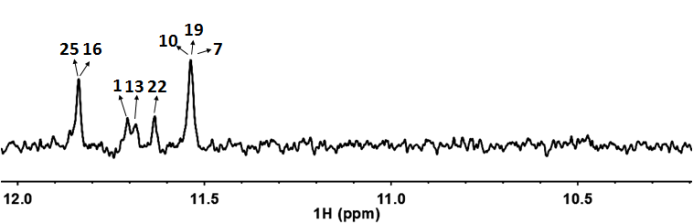


Fig S12.


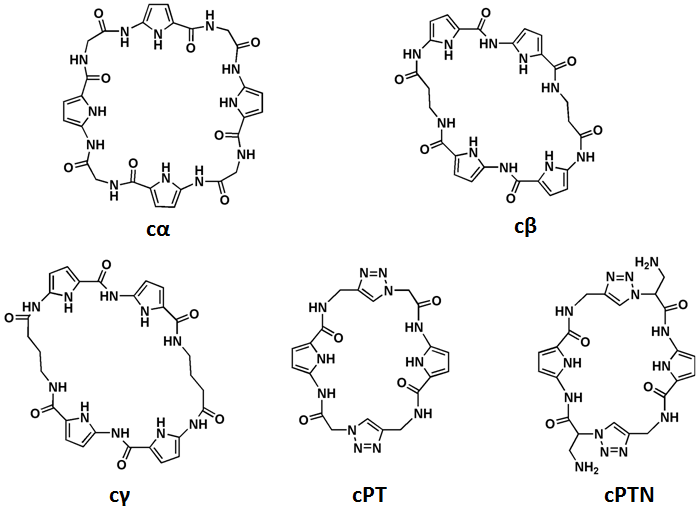


Fig S13.


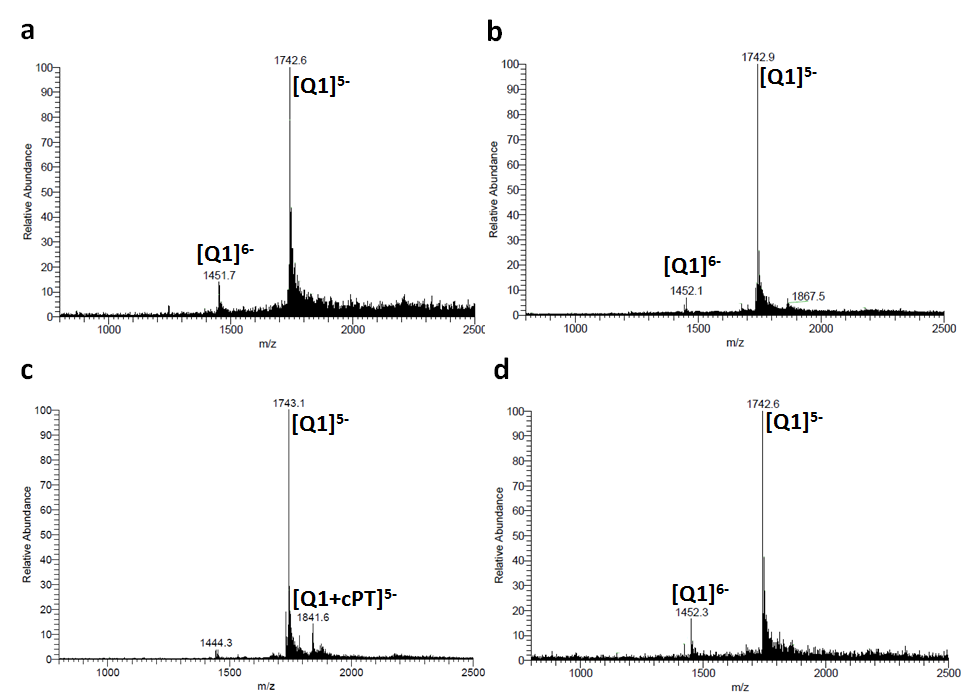


Fig S14.


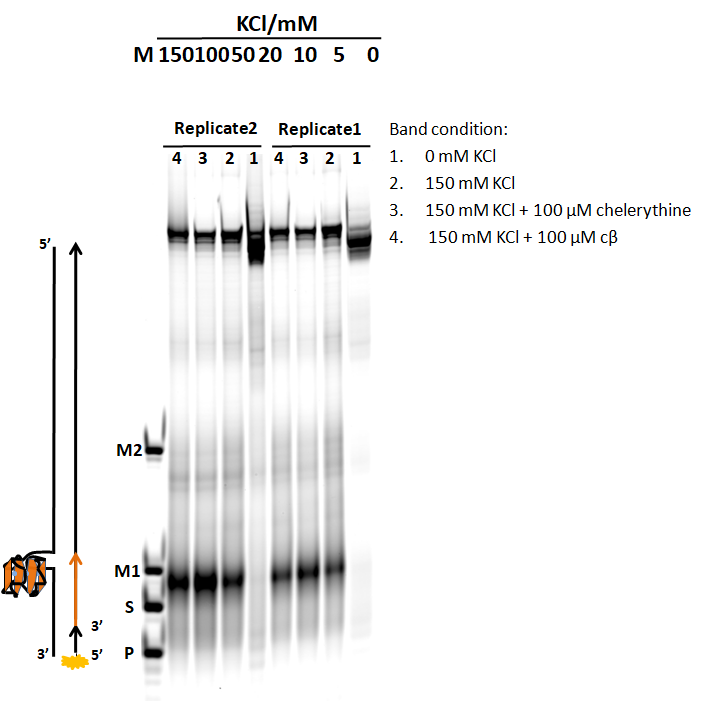

Supplement: Supplementary file 1 — Supporting Information [file 41598_2019_39941_MOESM1_ESM.docx]
